# Prescription Pattern of Chinese Herbal Products for Breast Cancer in Taiwan: A Population-Based Study

**DOI:** 10.1155/2012/891893

**Published:** 2012-05-28

**Authors:** Jung-Nien Lai, Chien-Tung Wu, Jung-Der Wang

**Affiliations:** ^1^Institute of Traditional Medicine, School of Medicine, National Yang-Ming University, Taipei City 112, Taiwan; ^2^Department of Chinese Medicine, Taipei City Hospital, Yangming Branch, Taipei City 111, Taiwan; ^3^Department of Chinese Medicine, Taipei City Hospital, Linsen Chinese Medicine Branch, Taipei City 104, Taiwan; ^4^Department of Public Health, College of Medicine, National Cheng Kung University, Tainan City 701, Taiwan; ^5^Departments of Occupational and Environmental Medicine and Internal Medicine, National Cheng Kung University Hospital, Tainan City 701, Taiwan

## Abstract

*Background*. Chinese herbal products (CHPs) given as a therapy for symptom relief have gained widespread popularity among women with breast cancer. The aim of this study was to analyze the utilization of CHP among women with breast cancer in Taiwan. *Methods*. The usage, frequency of services, and CHP prescribed for breast cancer among women with breast cancer were evaluated, recruited from a randomly sampled cohort of 1,000,000 beneficiaries from the National Health Insurance Research Database. The logistic regression method was employed to estimate the odds ratios (ORs) for utilization of CHP. *Results*. 81.5 percent (*N* = 2, 236) of women with breast cancer utilized traditional Chinese medicine (TCM) and 18% of them sought TCM with the intent of treating their breast cancer. Jia-wei-xiao-yao-san (*Augmented Rambling Powder*) was the most frequently prescribed formula for treating breast cancer. Among the top 10 most frequently prescribed CHP for treating breast cancer, seven contained dang qui (*Angelica sinensis-radix*) and six contained ren shen (*Panax ginseng-radix*), which are reported to have potential beneficial synergistic effects on breast cancer cells. *Conclusion*. CHP containing dang qui (*Angelica sinensis-radix*) or ren shen (*Panax ginseng-radix*) are the most frequently prescribed for breast cancer and their effects should be taken into account by healthcare providers.

## 1. Introduction

Despite the lack of solid evidence supporting their therapeutic benefit, the reported incidence of use of complementary and alternative medicines (CAMs) among women with breast cancer ranges from 66.7 to 97% [1–3]. An increased perception of breast cancer recurrence and of breast cancer-related death [4] may be the reasons why patients use a wide range of CAM including herbs, vitamins, homeopathic remedies, and Chinese herbal products (CHPs) [5–8]. The expectations of CAM use vary among individuals. Some just hope to strengthen their immune system, some expect to decrease the treatment-associated toxicity, and some want to alleviate the cancer-derived symptoms [9]. However, there is no compelling evidence supporting the effectiveness of CAM use in breast cancer patients [5, 10]. In view of such and without further knowledge on how effective CAM is, it is not easy for oncologists or CAM practitioners to provide an appropriate recommendation that can meet the expectations of women with breast cancer.

Traditional Chinese medicine (TCM) has been growing in popularity and has offered an important alternative or complement to health care in many countries. The most common type of CAM used by Chinese women with breast cancer is CHP [11]. Previous studies have disclosed the potential beneficial synergistic effects of the usage of *dang qui *(*Angelica sinensis-radix*) [12–14] or *ren shen *(*Panax ginseng-radix*) [15–17] among women with breast cancer. Although a previous four-year survey [11] indicated that over 50% of breast cancer patients considered CHP effective in treating cancer, the utilization of individual CHP has rarely been reported. In Taiwan, CHPs have been an important part of health care for hundreds of years and are fully reimbursed under the current National Health Insurance (NHI) system. Accordingly, the claims database provides a platform for understanding the utilization of CHP prescribed by licensed TCM doctors. The aim of our study is to analyze a random sample of this comprehensive database and to determine the CHP utilization patterns for women with newly diagnosed breast cancer in Taiwan. Results of this study may provide valuable information for physicians, enabling them to respond to patients' use of CHP in an informed way and strengthening the patient-physician relationship in breast cancer care.

## 2. Materials and Methods

### 2.1. Data Resources and Study Sample

This study was conducted after approval by the review board of the Committee on Chinese Medicine and Pharmacy (CCMP), Department of Health, Taiwan. It was designed as a population-based study to analyze a sample of 1 million subjects selected at random from the 22 million beneficiaries of the National Health Insurance scheme of Taiwan and to determine the prevalence of prescribed CHP in women with breast cancer between January 1, 1997, and December 31, 2008. All data were obtained from the National Health Insurance Research Database (NHIRD), which included all the reimbursement data of the NHI with identification numbers of all individuals encrypted, transformed, and maintained by the National Health Research Institutes of Taiwan [18]. The NHIRD database contained patient's gender and date of birth, all records of clinical visits and hospitalization, prescribed drugs and dosages, including CHP, and three major diagnoses coded in the *International Classification of Diseases, Ninth Revision, Clinical Modification* (ICD-9-CM) format [19].

**Figure 1:**
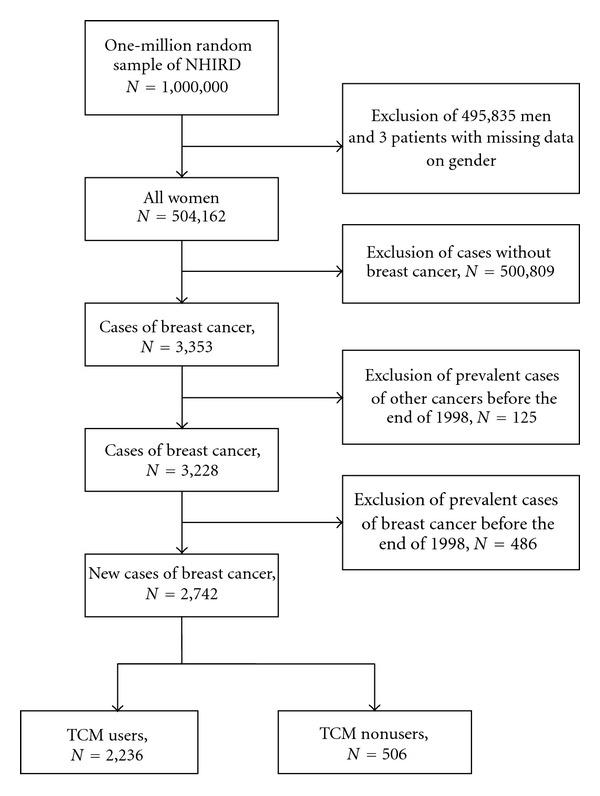
Flowchart of recruitment of subjects from the 1-million random sample of the National Health Insurance Research Database (NHIRD) from 1999 to 2008 in Taiwan.

The selection of study subjects from the random sample of one million individuals was performed as follows (Figure 1). First, we excluded all male beneficiaries (*n* = 495, 835) or those with missing information concerning gender (*n* = 3). Second, female beneficiaries without breast cancer (*n* = 500, 809) were excluded. Third, the prevalent cases of other cancers (*n* = 125) and breast cancer (*n* = 486) diagnosed before the end of 1998 were also excluded to make sure that all the subjects included were newly diagnosed with invasive breast cancer and the diagnosis was verified by the NHI registry of catastrophic illnesses during 1998–2008. All patients who are registered to have a catastrophic illness are exempted from all copayments. To be registered as such, patients must have the diagnosis of invasive breast cancer (*ICD-9 *code 174) validated by tissue pathology. Finally, 2,742 female subjects were included in the cohort.

### 2.2. Traditional Chinese Medicine

TCM treatments include CHP, acupuncture and manipulative therapies for trauma, all of which are reimbursed by the NHI of Taiwan. CHPs composed of one or more herbs (formula) are most widely adopted by patients in Taiwan [20]. To study the utilization of prescribed CHP in the present study, we downloaded the detailed herbal contents for all kinds of reimbursed CHP from the website of the CCMP, including the name of each CHP, the proportion of each constituent, the date and period of approval as drug, the code, and the name of manufacturer. For simplicity, all CHPs with the same CCMP standard formulas are classified under the same categories, regardless of slight variations among products of different pharmaceutical companies [21].

### 2.3. Study Variables

To determine the key independent variables for the utilization of CHP among women with breast cancer, we selected the demographic factors according to previous studies [22]. Ages were categorized into seven groups: ≤29, 30–39, 40–49, 50–59, 60–69, 70–79, and ≥ 80 years. Geographic areas of Taiwan were classified into the following six regions: Taipei city, Kaohsiung city, Northern, Central, Eastern, and Southern regions. We split the monthly wage into four levels: New Taiwan Dollars (NT$) 0, 1–19,999, 20,000–39,999, and ≥40,000.

### 2.4. Statistical Analysis

Data analysis was performed by descriptive statistics, including the prescription rates of CHP users stratified by patient's age, indications for the prescription of CHP, and the most frequently prescribed herbal formulas for treating breast cancer. Primary indications were classified according to the ICD-9. The diagnoses were coded according to the ICD-9 and grouped into different broader disease categories. For example, ICD-9 codes 460–519 were classified as diseases of the respiratory system, codes 780–799 were grouped as symptoms, signs, and ill-defined conditions, and codes 520–579 were classified as diseases of the digestive system. Multiple logistic regression was conducted to evaluate factors that correlated with CHP use. A significance level of *α* = 0.05 was selected. The statistical software SAS 9.13 was used for data management and analyses.

## 3. Results

The database of outpatient claims contained information on 2,742 women with breast cancer from 1999 to 2008. Among them, 2,236 (81.5%) breast cancer patients used TCM outpatient services. Most TCM users (95.8%) also received cancer treatment (Table 1). The mean age of TCM nonusers was significantly higher than that of TCM users. There were more TCM users than TCM nonusers with income level of NT$ 1–19,999 or residing in Central and Southern Taiwan. There was no significant difference in cancer treatment modalities between TCM users and non-TCM users.

Adjusted odds ratios (aORs) and 95% confidence intervals (95% CIs) obtained by multiple logistic regression are summarized in Table 1. Compared with the age group of 30–39 years (aOR = 1.00), there were no significant differences in ages between TCM users and TCM nonusers except those aged 80 years and above who were more likely to be non-TCM users. There was also no significant difference or trend among women in different income groups.

Chinese herbal medicines were prescribed in 22,755 (76.8%) of visits made by women to TCM doctors, with acupuncture and manipulative therapies for trauma prescribed for the rest. Analysis of the major disease categories for 2,236 TCM users (Table 2) showed that breast cancer was the most common reason for using CHP (21.7%, *n* = 6, 442), followed by “symptoms, signs, and ill-defined conditions” (17.5%, *n* = 5, 177), and “diseases of the respiratory system” (11.0%, *n* = 3, 253), as summarized in Table 2.

Details on the most frequently prescribed CHP for treating breast cancer by TCM doctors are provided in Table 3. As can be seen, *Jia-wei-xiao-yao-san *(*Augmented Rambling Powder*) is the most frequently prescribed CHP, followed by *Xiang-sha-liu-jun-zi-tang *(*Vladimiria and Amomum Combination*) and* Gui-pi-tang *(*Ginseng and Longan Combination*). Among the top 10 most frequently prescribed CHPs, seven containing *dang qui *(*Angelica sinensis-radix*) and six containing *ren shen *(*Panax ginseng-radix*) of various doses were identified.

Although over 81% (*n* = 2, 236) of women with breast cancer had used TCM and CHP as the major method of treatment from 1999 to 2008, only about 18% (*n* = 466) of them sought TCM with the intent of either treating their breast cancer or relieving the treatment-related side effects. CHP was prescribed in addition to surgery, radiation therapy, and/or chemotherapy and appeared to be used as an adjunct to conventional treatments for cancer, rather than as alternatives. The top three formulas most frequently prescribed by TCM doctors for treating breast cancer were *Jia-wei-xiao-yao-san *(*Augmented Rambling Powder*), *Xiang-sha-liu-jun-zi-tang *(*Vladimiria and Amomum Combination*), and* Gui-pi-tang* (*Ginseng and Longan Combination*).

## 4. Discussion

The increasing trend of TCM utilization among women with breast cancer in Taiwan is in line with trends reported from China [1]. However, the prevalence of CHP use to treat breast cancer among Taiwanese women is far below the proportions in other countries [2, 3, 6]. Previous studies reported that approximately 43–80% of breast cancer patients used CAM as part of the treatment for their breast cancer [1–3, 6]. Possibly fear of cancer recurrence and cancer-related death is motivation for women to use CAM therapies. The difference in results between the present study and those previously reported was probably due to the disparities in definition of breast cancer treatment between patients and licensed TCM doctors. Previous studies [1–3, 6] collected the information of breast cancer treatment via self-reported questionnaire, which represented the patients' own perception and expectation of the prescribed treatment. On the contrary, the perspective of TCM doctors concerning the treatment prescribed must be in line with the requirement of the NHI in Taiwan. They had to follow the standard diagnoses using the *ICD-9-CM *[20] coding system when claiming reimbursement, and women with breast cancer are exempted from all copayments once TCM doctors coded their diagnoses as ICD-9 code 174 (malignant neoplasm of female breast) to the NHI bureau. Another possible explanation is that the present study demonstrated only the utilization of CHP, which is the modern form of Chinese herbal remedies, of which a single herb and herbal formulas are concentrated into granulated compounds and made available like over-the-counter dietary supplements in the United States [23]. Decoctions and Chinese herbal remedies purchased directly from TCM herbal pharmacies, which were classified as Chinese herbal medications, were not included in the present study. Although the present findings cannot be generalized to the comprehensive usage of various types of CAM, the present study using a random national-level sample revealed the prevalence in use of CHP prescribed by licensed TCM doctors for treating breast cancer.

Although TCM as a unique traditional therapy for various ailments has been used in Taiwan for over hundreds of years, more than 95% of CHP users continued to receive standard breast cancer treatment during the 10-year study period. Moreover, regardless of the experiences caused by receiving different types of moderately toxic cancer treatments, the choice of major medical options among women with breast cancer was not associated with the use of CHP. Hence, we inferred that CHP for women with breast cancer in Taiwan was generally used as adjuncts to cancer treatment, rather than as replacements for it.

Previous clinical trials have demonstrated that *Jia-wei-xiao-yao-san *(*Augmented Rambling Powder*), which is the most frequently prescribed formula for treating breast cancer in Taiwan, may be an efficacious therapy for reducing psychological (anxiety and depression) symptoms in postmenopausal women [24]. Among the top 10 most frequently prescribed formulas for treating breast cancer, *Gui-pi-tang *(*Ginseng and Longan Combination*), *Tian-wang-bu-xin-dan *(*Ginseng and Zizyphus Combination*), and* Suan-zao-ren-tang *(*Zizyphus Combination*), which all have a long history of use, are said to nourish the blood and calm the nerves and are very often prescribed by TCM doctors to alleviate sleep disturbance [25]. Other commonly prescribed formulas are often for relieving gastrointestinal discomfort (*Ban-xia-xie-xin-tang*, *or Pinellia Combination*), poor appetite (*Xiang-sha-liu-jun-zi-tang*, *or Vladimiria and Amomum Combination*), fatigue (*Bu-zhong-yi-qi-tang*, *or Ginseng and Astragalus Combination*), palpitation (*Ren-shen-yang-rong-tang*, *or Ginseng Nutritive Combination*), or swelling of lymph nodes (*San-zhong-kui-jian-tang*, *or Forsythia and Laminaria Combination*). It is apparent from this study that TCM doctors in Taiwan prescribed herbal therapies mainly for reducing psychosocial distress and symptomatic discomfort. However, it remains to be clarified whether frequently prescribed CHPs containing* ren shen *(*Panax ginseng-radix*) and *dang qui *(*Angelica sinensis-radix*) for cancer treatment are intended by TCM doctors to decrease the treatment-associated toxicity or to alleviate the cancer-derived symptoms. Notably, there is yet insufficient evidence for reaching a conclusion regarding the cost-effectiveness of the simultaneous administration of cancer treatment with *dang qui *(*Angelica sinensis-radix*) or* ren shen *(*Panax ginseng Radix*) [26]. Further studies are warranted to assess *dang qui* (*Angelica sinensis-radix*) or* ren shen *(*Panax ginseng Radix*) as an add-on treatment for women receiving conventional breast cancer treatments.

Previous studies revealed that CHP users among women with breast cancer were more likely to have higher income or be of younger age [1]. However, the present data demonstrated no significant difference in these two variables, possibly, because the NHI system has a comprehensive coverage and the copayment for CHP is universally NT$50 (approximately US$1.5), which provides an affordable health access for all age groups and different income levels. Symptoms, signs, and ill-defined conditions and diseases of the respiratory system were the two most frequent diagnoses in disease category for TCM visits with prescription of CHP, after breast cancer. The results indicate that, besides breast cancer care, health care providers should pay more attention to the general health conditions of patients suffering from symptoms, signs, and ill-defined conditions as well as respiratory discomfort and provide proactive recommendations for these medical needs.

The present study has two limitations. First, because the identities of the patients were encrypted and thus not available in the NHI reimbursement database, we were unable to obtain any histopathology reports to verify the diagnoses. However, because the registration of breast cancer as a catastrophic illness is approved on the basis of pathology and/or cytology evidence and is followed by a full waiver of copayment, such a diagnosis is made only after very serious review and is generally accurate. The diagnostic accuracy of breast cancer among the NHI data is corroborated by the significant agreement between the incidence rate calculated herein and that determined by the National Cancer Registry of Taiwan, in which 95% of the breast cancers are accompanied by histopathologic validation. Second, this study did not include Chinese herbal remedies purchased directly from TCM herbal pharmacies, nor did we include health food containing herbs. Thus, the frequency of CHP utilization might be underestimated. However, because the NHI system has a comprehensive coverage for TCM prescriptions, which is generally less than the cost of herbs sold in Taiwan markets, the likelihood that subjects purchased lots of other herbs outside the NHI database is not high.

## 5. Conclusions

It is apparent that our findings may have implications for physicians attending to women with breast cancer. Our results suggest that, under the coexistence of the conventional medical treatments and TCM, most breast cancer patients consumed herbal therapies with the intention of relieving their treatment-induced symptoms, rather than rejecting standard cancer treatments. Recognizing the use of TCM, exploring potential interactions and adverse effects, and integrating both technologies may be more beneficial to the overall health, or survival and quality of life, of breast cancer patients. Thus, health care providers had better proactively explore a personalized optimal treatment for breast cancer, as well as attend to the patients' psychosocial and physical needs.

## Figures and Tables

**Table 1:** Demographic characteristics and results of multiple logistic regression showing the adjusted odds ratio (aOR) and 95% CI (confidence interval) of women with newly diagnosed breast cancer from the 1-million random sample of the National Health Insurance Research Database (NHIRD) from 1999 to 2008 in Taiwan.

Characteristics	TCM^a^ nonusers	TCM users	aOR^b^ (95% CI^c^)
No. of cases	506	2,236	
CHP^d^ for breast cancer	—	503	
Age at diagnosis (years)			
≤29	8	26	0.63 (0.26–1.52)
30–39	51	283	1
40–49	165	771	0.84 (0.59–1.19)
50–59	146	617	0.78 (0.54–1.11)
60–69	75	356	0.86 (0.57–1.28)
70–79	40	146	0.75 (0.46–1.22)
≥80	21	37	0.35 (0.18–0.68)
Insured salaries (NT$^e^/month)			
0^+^	120	416	1
1–19999	234	1178	1.24 (0.93–1.64)
20000–39999	96	432	1.02 (0.73–1.43)
>40000	56	210	0.96 (0.65–1.42)
Insured region			
Taipei city	171	555	1
Kaohsiung city	34	148	1.33 (0.88–2.02)
Northern Taiwan	159	644	1.27 (0.98–1.63)
Middle Taiwan	49	380	2.66 (1.86–3.79)
Southern Taiwan	80	431	1.83 (1.33–2.52)
Eastern Taiwan	10	59	2.25 (0.98–5.14)
Cancer treatment modalities			
No treatment	26	93	0.86 (0.49–1.51)
Surgery only	46	207	1
Chemotherapy only	7	29	0.98 (0.39–2.47)
Hormone therapy only	21	98	1.07 (0.60–1.91)
Surgery and chemotherapy	74	305	0.82 (0.54–1.25)
Surgery and hormone therapy	113	508	1.10 (0.74–1.63)
Surgery, chemotherapy, and hormone therapy	193	885	1.00 (0.69–1.44)
Others	26	111	0.99 (0.60–1.63)

^a^TCM refers to traditional Chinese medicine; ^b^OR refers to odds ratio; ^c^CI refers to confidence interval; ^d^CHP refers to Chinese herbal products;^e^ NT$ refers to new Taiwan dollars, of which 1 US$ = 30 NT$.

**Table 2:** Frequency distribution of traditional Chinese medicine (TCM) visits by major disease categories (according to 9th ICD codes) in women with breast cancer from 1999 to 2008 in Taiwan.

Major disease category	*ICD-9-CM codes *	No. of visits *N* (%)		
		Chinese herbal remedies	Acupuncture and manipulative therapies	Total of TCM
Infectious and parasitic diseases	001–139	44 (0.1)	3 (0.0)	47 (0.2)
Neoplasms	140–239	6,672 (22.5)	1,165 (3.9)	7,837 (26.5)
Breast cancer	174	6,442 (21.7)	1,145 (3.9)	7,587 (25.6)
Other cancers (remainder of neoplasms)		230 (0.8)	20 (0.1)	250 (0.8)
Endocrine, nutritional and metabolic diseases, and immunity disorders	240–279	262 (0.9)	1 (0.0)	263 (0.9)
Mental disorders	290–319	127 (0.4)	9 (0.0)	136 (0.5)
Diseases of the nervous system and sense organs	320–389	446 (1.5)	69 (0.2)	515 (1.7)
Diseases of the circulatory system	390–459	310 (1.0)	17 (0.1)	327 (1.1)
Diseases of the respiratory system	460–519	3,253 (11.0)	64 (0.2)	3,317 (11.2)
Diseases of the digestive system	520–579	2,222 (7.5)	72 (0.2)	2,294 (7.7)
Diseases of the genitourinary system	580–629	1,294 (4.4)	74 (0.2)	1,368 (4.6)
Diseases of the skin and subcutaneous tissue	680–709	366 (1.2)	12 (0.0)	378 (1.3)
Diseases of the musculoskeletal system and connective tissue	710–739	1,681 (5.7)	2,461 (8.3)	4,142 (14.0)
Symptoms, signs, and ill-defined conditions	780–799	5,177 (17.5)	108 (0.4)	5,285 (17.8)
Injury and poisoning	800–999	640 (2.2)	2,736 (9.2)	3,376 (11.4)
Supplementary classification^d^	V01–V82, E800–E999	41 (0.1)	59 (0.2)	100 (0.3)
Others*		220 (0.7)	23 (0.1)	243 (0.8)

Total		22,755 (76.8)	6,873 (23.2)	29,628 (100)

*Others include ICD-9-CM codes 280–289, *630*–*677, 740–759, *760*–*779 and missing/error data.

^d^Supplementary classification of factors influencing health status and contact with health service, external causes of injury and poisoning.

**Table 3:** Top 10 herbal formulas prescribed by TCM doctors for treating breast cancer among 503 breast cancer women from 1999 to 2008 in Taiwan.

Herbal formulas	English name	Frequency of prescriptions *N* = 6,442 (%)	Average daily dose (g)	Average duration for prescriptions (day)
*Jia-wei-xiao-yao-san* ^†^	Augmented Rambling Powder	1,045 (16.2)	5.1	12.8
*Xiang-sha-liu-jun-zi-tang* ^‡^	*Vladimiria* and *Amomum* Combination	450 (7.0)	5.5	11.5
*Gui-pi-tang* ^†‡^	*Ginseng* and *Longan* Combination	414 (6.4)	5.0	12.1
*San-zhong-kui-jian-tang* ^†^	*Forsythia* and *Laminaria* Combination	367 (5.7)	6.3	10.2
*Bu-zhong-yi-qi-tang* ^†‡^	*Ginseng* and *Astragalus* Combination	347 (5.4)	5.2	12.3
*Tian-wang-bu-xin-dan* ^†‡^	*Ginseng* and *Zizyphus* Combination	302 (4.7)	4.7	13.8
*Ban-xia-xie-xin-tang* ^‡^	*Pinellia* Combination	289 (4.5)	5.0	11.6
*Suan-zao-ren-tang *	*Zizyphus* Combination	276 (4.3)	4.5	13.4
*Ren-shen-yang-rong-tang* ^†‡^	*Ginseng* Nutritive Combination	272 (4.2)	5.1	10.3
*Xue-fu-zhu-yu-tang* ^†^	*Persica* and *Achyranthes* Combination	254 (3.9)	3.8	12.2

^†^Chinese herbal products containing *dang qui (Angelica sinensis-radix).*

^‡^Chinese herbal products containing *ren shen (Panax ginseng-radix).*
